# Pexidartinib Inhibits Macrophage Senescence Through Glycolysis in Periodontitis Microenvironment

**DOI:** 10.1016/j.identj.2025.100843

**Published:** 2025-06-02

**Authors:** Jifan Zhan, Jiabing Kang, Yan Wei, Tianjiao Xiao, Hui Fang, Li Li, Yiting Yuan, Yongchun Zhang, Jie Zhang, Ai Tian

**Affiliations:** a The Stomatology of Guizhou Medical University, Guizhou Medical University, Guiyang, China; b Postgraduate Research Institute, Kunming Medical University, Kunming, China; c Department of Experimental Orofacial Medicine, Philipps-University Marburg, Marburg, Germany; d The First Affiliated Hospital of Guizhou University of Chinese Medicine, Guiyang, China; e The Affiliated Stomatological Hospital & Stomatology of Guizhou Medical University, Guizhou Medical University, Guiyang, China

**Keywords:** Periodontitis, Macrophage, Cellular senescence, Glycolysis, Colony-stimulating factor-1 receptor (CSF-1R), Pexidartinib

## Abstract

**Objectives:**

Periodontitis, a chronic inflammatory condition, is caused by complex interactions between periodontopathic bacteria and the local innate immune response. Macrophage senescence, a pivotal contributor to immune dysfunction, has been implicated in periodontitis progression. This research was conducted to clarify how macrophage senescence and glycolysis interact within the periodontal inflammatory microenvironment. Specifically, we investigated whether pexidartinib (PLX3397), known for inhibiting the colony-stimulating factor-1 receptor (CSF-1R), could mitigate macrophage senescence by modulating glycolytic activity, thereby attenuating periodontal inflammation.

**Methods:**

We first constructed the experiential periodontitis mouse model. The alveolar bone volume was quantified using Micro-CT, while the periodontal ligament width and the distance from the cementoenamel junction (CEJ) to the alveolar bone crest (ABC) were evaluated using HE staining. The expression levels of macrophage senescence markers, glycolysis-related indicators, and CSF-1R in gingival tissues were assessed by immunofluorescence staining. For in vitro studies, Senescence was induced in RAW264.7 cells by stimulating them with *Porphyromonas gingivalis* lipopolysaccharide (*Pg*-LPS) with or without pretreatment of PLX3397 and glycolysis modulators. Cellular senescence rates were evaluated using Senescence-associated β-Galactosidase (SA-β-Gal) staining. The presence of markers indicating senescence, CSF-1R, and glycolysis-related indicators was further analysed by RT-qPCR and Western blotting.

**Results:**

The gingival tissues of mice with periodontitis showed elevated senescent macrophages, which correlated with higher CSF-1R expression and glycolytic activity. Similarly, in *Pg*-LPS-treated RAW264.7 macrophages, senescence markers were upregulated alongside CSF-1R and glycolysis-related indicators. Meanwhile, modulating glycolysis in vitro directly influences senescence indicators. And PLX3397 treatment reduced glycolytic activity, leading to an improvement in macrophage senescence.

**Conclusion:**

Our findings indicate that PLX3397 alleviates periodontal tissue inflammation by inhibiting macrophage senescence via glycolytic modulation, offering potential for immune-regulatory therapies in periodontitis management.

## Introduction

Periodontitis is a common long-term inflammatory condition resulting from complex interactions between periodontal pathogens and local immune responses.^1^ Globally, it ranks as the sixth most common ailment, affecting 50% to 60% of the population.^2^ Increasing evidence highlights the critical role of senescent cells in driving chronic inflammation in periodontitis.^3^ Senescent cells accumulate and propagate senescence to neighbouring cells, creating a synergistic effect. Senescence-associated inflammation brought on by the buildup of senescent cells and bacteria-induced inflammation both contribute to a "two-source periodontal inflammation" model, thereby exacerbating disease progression.^4^ Targeting senescent cells hasbecome a potential approach for treating periodontitis.^3^^,^^5^, ^6^, ^7^

Cellular senescence, which involves permanent cell cycle arrest and the Senescence-Associated Secretory Phenotype (SASP), disrupts tissue homeostasis by amplifying local inflammation and impairing regeneration.^8^ SASP factors, primarily secreted by senescent macrophages, propagate inflammation via autocrine and paracrine pathways.^9^ Macrophages, which are key carriers of SASP, are terminally differentiated cells.^10^ Although macrophages do not undergo replicative senescence, they can experience stress-induced senescence triggered by bacterial toxins (such as LPS) or oxidative stress.^11^^,^^12^ Our previous work demonstrated that LPS-induced senescence in macrophages promotes SASP secretion, exacerbating periodontal inflammation.^13^ Thus, strategies targeting senescent macrophages or SASP inhibition represent precise immunomodulatory approaches for periodontal inflammatory progression.

Colony-stimulating factor-1 receptor (CSF-1R), belonging to the class III receptor tyrosine kinases,^14^ is predominantly expressed on macrophages and osteoclasts.^15^ It drives osteoclastogenesis and pro-inflammatory cytokine production,^16^ contributing to skeletal and inflammatory diseases. Therefore, the inhibition of CSF-1R is seen as a possible therapeutic approach to block inflammation and bone loss. Elevated expression of CSF-1R in macrophages mediates the release of inflammatory factors and drives inflammatory alveolar bone resorption, contributing to the pathological progression of periodontitis.^17^ Blocking CSF-1R, particularly using the inhibitor pexidartinib (PLX3397), has shown potential in alleviating inflammation in periodontal tissues.^17^, ^18^, ^19^ Our preliminary findings indicate that PLX3397 mitigates LPS-induced macrophage senescence by downregulating senescence markers and SASP factors,^13^ though the underlying mechanisms remain unclear.

Emerging evidence suggests that CSF-1R influences cell metabolic reprogramming, particularly glycolysis.^20^^,^^21^ Glycolysis, a key metabolic pathway, dynamically regulates macrophage activation and function.^22^^,^^23^ In addition, aberrant glycolytic activity is closely associated with senescence.^24^, ^25^, ^26^ Studies have shown that LPS enhances glycolytic activity in macrophage,^9^ and induces macrophage senescence.^12^ Existing research collectively establish a close link between inflammation, immunity, and glucose metabolism,^27^ suggesting that inhibiting macrophage glycolysis may serve as a strategy to modulate cellular senescence and pro-inflammatory secretion.

This study aims to explore whether PLX3397 modulates macrophage senescence by targeting glycolysis in the periodontal inflammatory microenvironment. In this study, we initially assessed the association between macrophage senescence, glycolysis, and CSF-1R in periodontitis using animal experiments. In further mechanistic investigations, we employed *Porphyromonas gingivalis* LPS (*Pg*-LPS) to induce stress-induced senescence in RAW264.7 cells, mimicking macrophage senescence within the periodontal inflammatory microenvironment, and to explore whether PLX3397 could decelerate macrophage senescence and decrease the release of pro-inflammatory factors by regulating glycolysis. Our findings provide experimental evidence supporting novel immune-regulatory strategies for treating periodontitis.

## Methods

### Mouse model of experimental periodontitis

All procedures involving animals were conducted with the Animal Ethics Committee at Guizhou Medical University (Approval: 2200991) and adhered to institutional and ARRIVE 2.0 guidelines. In the experiment, twelve specific pathogen-free (SPF) male C57BL/6 mice (7 weeks old, body weight 25 ± 5 g) provided by the Animal Experiment Center of Guizhou Medical University were used. All mice were bred and maintained under standard conditions at the Animal Experiment Center of Guizhou Medical University, with unlimited access to food and water, and they were acclimated for a week beforehand. Two groups of mice were formed at random: control (N, n = 6) and periodontitis (PDs, n = 6). Periodontitis was induced under tribromoethanol anesthesia (0.2 mL/10 g; Nanjing Aibei Biotech, China, M2910) by tying a 5-0 silk ligature on the mandibular first molar for 2 weeks.^28^^,^^29^ Surgical procedures were performed under strict aseptic conditions to minimise nonligature-related infections.

### Micro-computed tomography imaging and analysis

Mandibular jaws were collected, and soft tissues were carefully removed. Samples were scanned using a SkyScan 1276 micro-CT scanner (Bruker, Germany) with the following parameters: 85 kV X-ray voltage, 200 μA current, 6.53 μm voxel size, 384 ms exposure time, Al 1mm filter, and 0.3° rotation step over 180°. Three-dimensional reconstructions were generated using NRecon software (version 1.7.4.2), with the region of interest being the alveolar bone surrounding the mandibular first molar.^1^^,^^30^, ^31^, ^32^

### HE staining

Mandibles were decalcified in 10% ethylenediaminetetraacetic acid (EDTA) over the course of 3 weeks, with solutions refreshed every 48 h. Decalcified, paraffin-embedded, sectioned, then dyed using hematoxylin and eosin (HE) were performed on the samples. Morphological analysis was conducted using light microscope images.

### Immunofluorescence staining

Mandibular specimens were prepared for paraffin embedding and sectioning after being fixed in 4% paraformaldehyde and decalcified with EDTA. Three nonconsecutive tissue sections per animal (N and PDs groups) were analysed to ensure representative sampling. Serial sections were subjected to immunofluorescence staining with these combinations: F4/80, p16 and CSF-1R; F4/80, p16 and GLUT1. Sections were incubated with primary antibodies against F4/80 (1:1,000, Abcam, UK, Ab300421), CSF-1R (1:200, Immunoway, USA, YT0881), p16 (1:200, HUABIO, China, SR34-02), and GLUT1 (1:200, Immunoway, USA, YT1928). After washing, samples were treated with fluorescently labeled secondary antibodies and counterstained with DAPI for nuclear visualisation. The images were captured using microscopy, and triple-positive cells were quantified using ImageJ software (v1.53). For area normalisation, the total cell count per sample was divided by the cumulative tissue area of all analysed fields to calculate cells/mm².

### Cell culture

The RAW264.7 cell line belongs to the macrophage cell line and was purchased from Shanghai Fuheng Biotechnology Company Limited (FuHeng BioLogy, China, FH0328). RAW264.7 macrophages were cultured in high-glucose Dulbecco's Modified Eagle’s Medium supplemented with 10% fetal bovine serum at 37°C in a humidified incubator with 5% CO₂. For inflammation modeling, cells were exposed to 1 μg/mL *Pg-*LPS (Invivogen, France, Tlrl-Pglps) for a duration of 24 hours.^12^^,^^13^^,^^33^ For PLX3397 treatment, cells were pre-incubated with 500 nM PLX3397 (MedChemExpress, China, HY-16749) for 4 hours before *Pg-*LPS stimulation. Glycolysis inhibition and enhancement were achieved using 1 mM 2-DG (MCE, USA, HY-13966) ^34^ and 100 ng/mL GM-CSF (PeproTech, USA, 315-03),^35^ respectively, and then stimulated with *Pg*-LPS as mentioned above. Total mRNA and protein were harvested after 24 hours.

### Senescence-associated-β-Galactosidase staining

SA-β-Gal staining was detected using a commercial kit (Beyotime Biotech, China, C0602) following the instructions provided by the manufacturer.After 15 minutes of fixation in 4% paraformaldehyde, the cells were stained and left in the dark at 37°C overnight. Senescent cells are identified by blue coloration under a microscope.

### Reverse transcription quantitative polymerase chain reaction (RT-qPCR)

After induction under varying conditions, total RNA was isolated from RAW264.7 cells using the RNA-Quick Purification Kit (ESscience Biotech, China, RN001) and subsequently converted into cDNA. ChamQ Universal SYBR qPCR Master Mix (Vazyme Biotech, China, Q711) was used to analyse SASP factors and glycolysis markers. GAPDH served as the internal control, and the relative expression of genes was assessed via the 2^−^*^ΔΔCT^* method. Control group data are presented as normalised fold change = 1 without error bars, as per the 2^−ΔΔ^*^CT^* method.^36^ Error bars for experimental groups represent SD from biological and technical replicates.

The sequences for the RT-qPCR primers are provided in Table 1.

**Table 1:** Primer sequences used for RT-qPCR.

Gene	Sequences of probes (5′-3′)	Length (bp)	Product (bp)
GAPDH	F:GGTTGTCTCCTGCGACTTCA	20	183
	R:TGGTCCAGGGTTTCTTACTCC	21	
IL-6	F:CTTCTTGGGACTGATGCTGGT	21	94
	R:AGGTCTGTTGGGAGTGGTATCC	22	
IL-1β	F:AGCTTCAGGCAGGCAGTATC	20	77
	R:AAGGTCCACGGGAAAGACAC	20	
TNF-α	F:ATGAGCACAGAAAGCATGATC	21	276
	R:TACAGGCTTGTCACTCGAATT	21	
GLUT1	F:ATGGATCCCAGCAGCAAGAAGG	22	80
	R:CCGAACTGCAGTGATCCGAG	20	
HK2	F:GTTTCTCTATTTGGCCCCGACT	22	117
	R:TGGTAGAGATACTGGTCAACCTTC	24	

### Western blotting

The BCA protein assay kit (Beyotime Biotech, China, P0012) was used to quantify the concentration of protein extracted from RAW264.7 cell lysates. Protein was isolated on 10% SDS-PAGE gels and then put on membranes made of PVDF. The membranes were blocked for 1 hour at room temperature in 5% skimmed milk prepared with TBST, followed by an overnight incubation at 4°C using primary antibodies specific for β-actin (1:10,000, Immunoway, USA, YM3028), CSF-1R (1:1,500, Immunoway, USA, YT0881), GLUT1 (1:1,000, Immunoway, USA, YT1928), HK2 (1:1,000, HUABIO, China, HA500186), p16 (1:1,500, HUABIO, China, SR34-02), and p21 (1:1,500, HUABIO, China, HA500005). After washing, membranes were cleaned and then incubated for one hour at ambient temperature with secondary antibodies (1:5,000, HUABIO, China, HA1006). The identification of protein bands was done with ECL reagents, and ImageJ software was used for quantification.

### Bioinformatics analysis

Gene expression data related to periodontal disease were retrieved from the GEO repository (https://www.ncbi.nlm.nih.gov/geo)under the dataset identifier GSE10334.^37^ Demographic and clinical characteristics of the cohort (including age, gender, race, ethnicity, and periodontal severity) are summarised in Supplementary Table 1. The inclusion criteria for dataset selection were as follows: (1) gene expression profiles generated using microarray technology; (2) PD datasets containing samples from gingival tissues; (3) inclusion of unaffected control samples; and (4) samples derived from Homo sapiens. GSE10334 was created using the GPL570 [HG-U133_Plus_2] Affymetrix Human Genome U133 Plus 2.0 Array platform. As the dataset is publicly accessible, this study did not require ethics committee approval or informed consent. Data analysis was performed using R software (version 4.1.1) with the "limma" R package.^38^ Data were first normalised using the normalise between arrays function. Probe IDs were then converted into official gene symbols. Significant differentially expressed genes (DEGs) were determined by computing the log2 fold change and adjusted p-value for each gene using the lmFit and eBayes functions, with criteria of |log2 fold change| > 1 and adjusted *P* < .05.

### Statistical analysis

ImageJ software (v1.53) was used for image analysis, and each experiment was conducted 3 times. With the help of GraphPad Prism 9.0, the data were examined and shown as mean ± SD (Standard Deviation). Normality was assessed using the Shapiro-Wilk test. One-way ANOVA was used for group comparisons, followed by Tukey's post hoc test. Statistical significance is defined as *P* < .05.

## Results

### Macrophage senescence is accompanied by enhanced CSF-1R expression in the periodontal inflammatory microenvironment

**Fig. 1:**
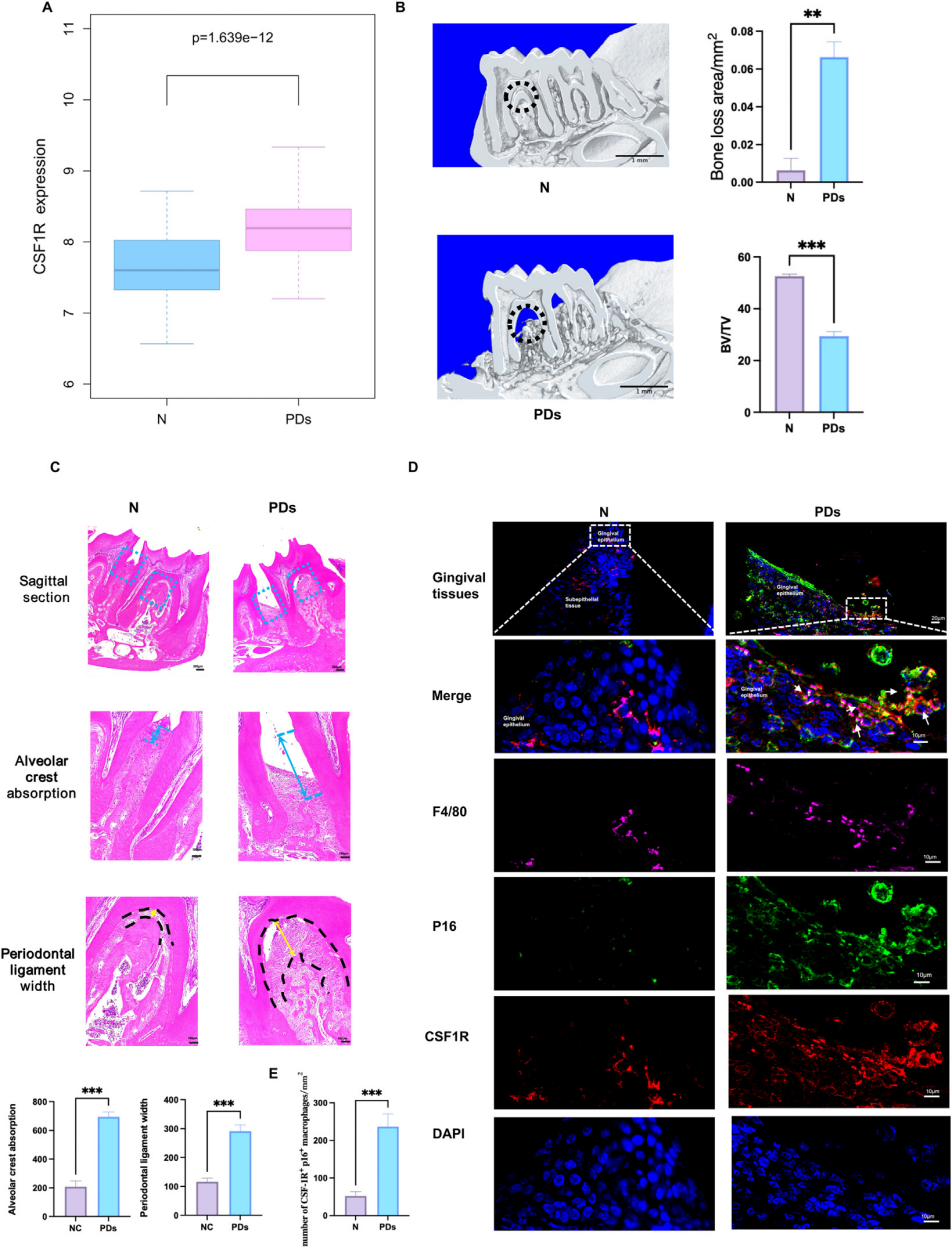
Macrophage senescence is accompanied by enhanced CSF-1R expression in vivo. A, Box plot of CSF-1R expression levels from the GSE10334 dataset. B, Three-dimensional micro-CT reconstruction pictures of the alveolar bone of the first molar. Quantitative examination of bone loss area and BV/TV in molar furcations was shown by bar histograms. Scale bar, 1 mm. C, HE staining of the gingival epithelium. The blue arrow indicates the distance of ECJ-ABC. The yellow arrow indicates the width of the periodontal membrane. Scale bar, 200 and 100 μm. Bar histograms illustrated measurements of alveolar crest resorption and the width of the periodontal ligament. Bars:200 μm and 100 μm. D, Immunofluorescence staining was performed on gingival tissues from the N and PDs groups using an F4/80 antibody to label macrophages (pink), a p16 antibody (green) and a CSF-1R antibody (red). The nuclei were stained with DAPI (blue). White arrows point to the triple-positive cells. E, Quantification of CSF-1R+ p16+ F4/80+cells. Bars: 20 μm and 10 μm.N, normal mice; PDs, periodontitis mice. Data were represented as the mean ± SD (n = 3) and analysed relative to normal mice. *P < .05, **P < .01, ***P < .001, ****P < .0001 vs N

CSF-1R is essential for macrophage chemotaxis, proliferation, and polarisation. Bioinformatics analysis of the GSE10334 dataset demonstrated that CSF-1R is significantly upregulated in periodontitis compared to controls (Figure 1A). According to the box plot, the periodontitis group (PDs) exhibits increased CSF-1R expression relative to the normal control group (N), highlighting its potential role in periodontal inflammation. To investigate the relationship between macrophage senescence and CSF-1R, we established a murine periodontitis model via molar ligation. Micro-CT analysis revealed that alveolar bone resorption at the furcation area of the mandibular first molar was markedly more pronounced in the periodontitis group than in the normal control group (Figure 1B). The PDs group demonstrated structural destruction and enlarged periodontal ligament spaces, as revealed by HE staining, in contrast to the N group. (Figure 1C). In addition, compared to the N group, the CEJ to the ABC distance at the distal root of the first molar and the mesial root of the second molar was markedly increased in the PDs group, indicating substantial alveolar crest resorption (Figure 1C). These findings validate the effective establishment of the experimental periodontitis model.

To examine the relationship between CSF-1R and macrophage senescence, we performed triple immunofluorescence staining for F4/80 (macrophages), p16 (senescence marker), and CSF-1R. In comparison to the N group, the PDs group exhibited a notable increase in the number of CSF-1R^+^ p16^+^ F4/80^+^ triple-positive cells in gingival tissues (Figure 1D, E). These findings indicate that senescent macrophages in periodontal tissues are associated with elevated CSF-1R expression in periodontitis mice.

**Fig. 2:**
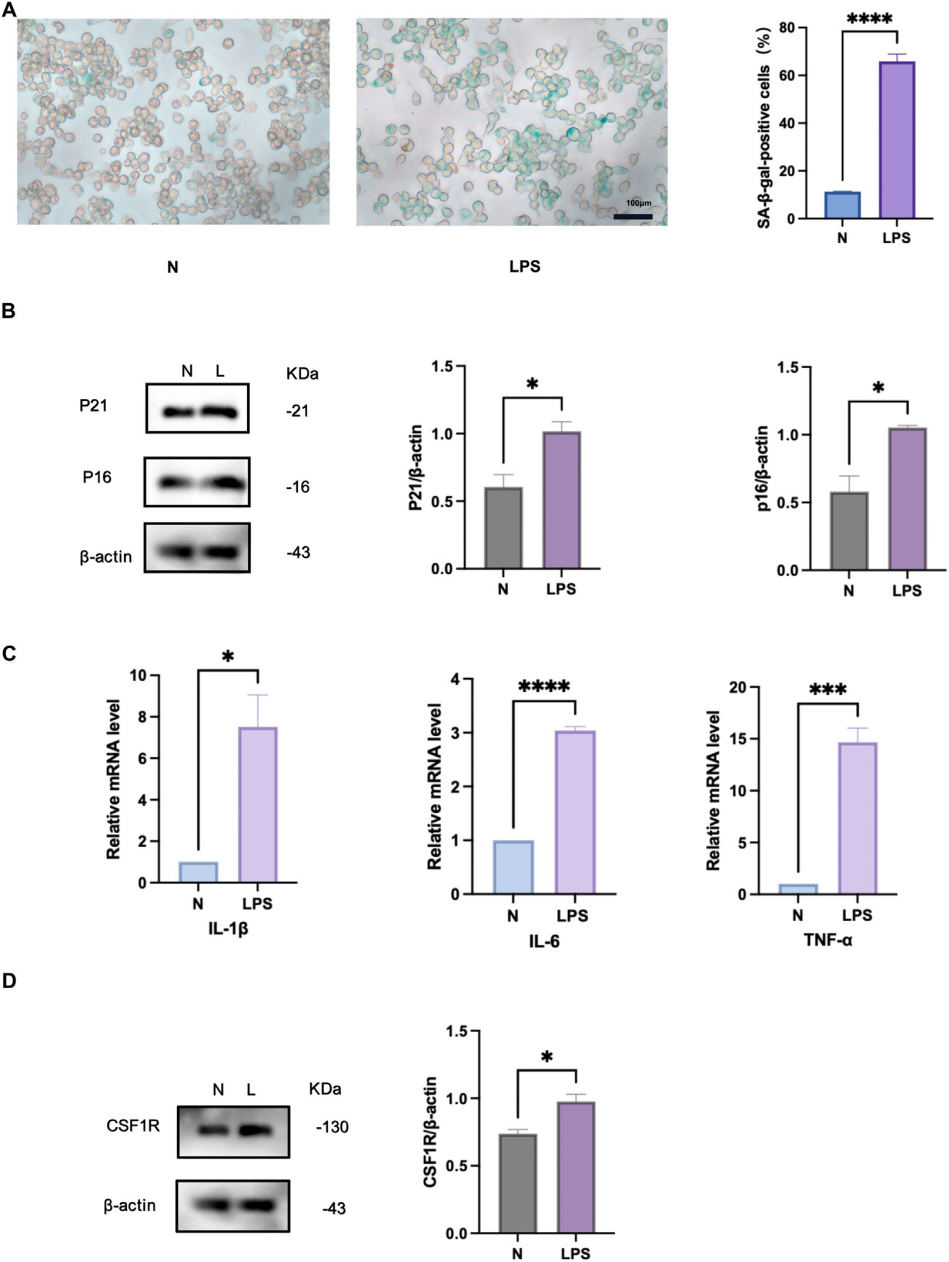
RAW264.7 cell senescence is accompanied by enhanced CSF-1R expression in vitro. A, The senescent cells were visualised using SA-β-Gal staining. Senescence in RAW264.7 cells was symbolised by the blue. Scale bar, 100 μm. B, Effect of *Pg*-LPS on the expression of p16,p21 protein in RAW264.7 cell. C, Impact of *Pg*-LPS on the expression of SASP factors mRNA in RAW264.7 cell. D, The impact of *Pg*-LPS on RAW264.7 cell CSF-1R protein expression. N, normal mice; PDs, periodontitis mice; N, control cells; L/LPS, RAW264.7 cells cultured in *Pg*-LPS(1μg/ml) for 24 hours. Data were represented as the mean ± SD (n = 3) and analysed relative to the control group, *P < .05, **P < .01, ***P < .001, ****P < .0001 vs N

To further characterise macrophage senescence under periodontal inflammatory conditions, we stimulated RAW264.7 cells with *Pg*-LPS (1 μg/mL, 24 hours) in vitro to mimic stress-induced senescence. Senescent cells were identified by an increased rate of cells positive for SA-β-Gal staining, an important marker of cellular senescence. The senescence-associated cell cycle suppressor proteins p16 and p21 are used as biomarkers and effectors of senescence, while IL-6, IL-1β, and TNF-α, were evaluated to characterise the SASP-associated factors.^3^ *Pg*-LPS stimulation induced distinct features of senescence in RAW264.7 cells. In comparison to the control group, *Pg*-LPS-treated cells exhibited elevated SA-β-Gal staining (Figure 2A), and increased protein expression of p16 and p21 (Figure 2B), and upregulated mRNA levels of SASP factors (Figure 2C). Additionally, Western blotting revealed a significant increase in CSF-1R protein expression following *Pg*-LPS treatment (Figure 2D). These results indicated that *Pg*-LPS stimulation promoted macrophage senescence. Furthermore, the findings demonstrate a connection between CSF-1R expression and macrophage senescence within the periodontal inflammatory microenvironment, as observed in vivo.

### PLX3397 inhibited *Pg*-LPS-induced RAW264.7cell senescence

**Fig. 3:**
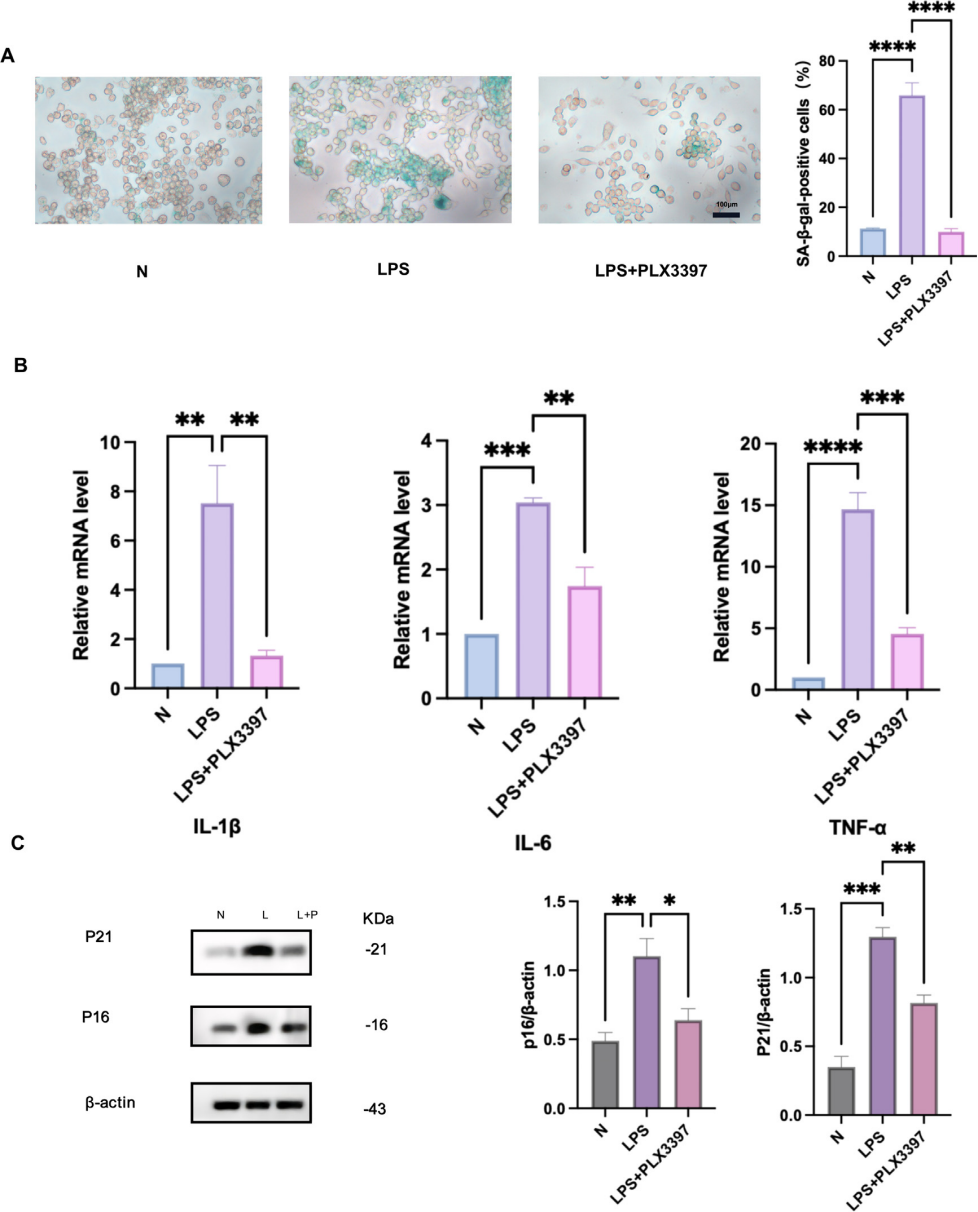
PLX3397 Inhibited *Pg*-LPS-induced senescence in RAW264.7 cell. A. The senescent cells were visualised using SA-β-Gal staining. Senescence in RAW264.7 cells was symbolised by the blue. Scale bar, 100 μm. B. Effect of PLX3397 pretreatment on the expression of SASP factor mRNA in RAW264.7 cells stimulated by *Pg*-LPS. C. The pretreatment of PLX3397 influences the expression of p16 and p21 proteins in *Pg*-LPS-stimulated RAW264.7 cells. N, control cells; L/LPS, RAW264.7 cultured in *Pg*-LPS(1μg/ml) for 24h, L+P/LPS+PLX3397 was 500nM PLX3397 pretreatment group. Data were represented as the mean ± SD (n=3) and analysed relative to the *Pg*-LPS group, *P < .05, **P < .01, ***P < .001, ****P < .0001vs. LPS.

PLX3397, known for inhibiting CSF-1R, has demonstrated therapeutic potential in attenuating periodontitis progression. To explore its effects on macrophage senescence, RAW264.7 cells were pretreated with 500 nM PLX3397 prior to 24-hour stimulation with 1 μg/mL *Pg*-LPS. Compared to the *Pg*-LPS group, PLX3397 reduced SA-β-Gal activity (Figure 3A). Additionally, the protein expression of senescence markers p16 and p21, as well as the mRNA expression of SASP factors(IL-1β, IL-6, and TNF-α), were significantly decreased (Figure 3B, C). These findings suggest that PLX3397 effectively mitigates macrophage senescence in RAW264.7 cells.

### Macrophage senescence accompanied by increased glycolysis in the periodontal inflammatory microenvironment

**Fig. 4:**
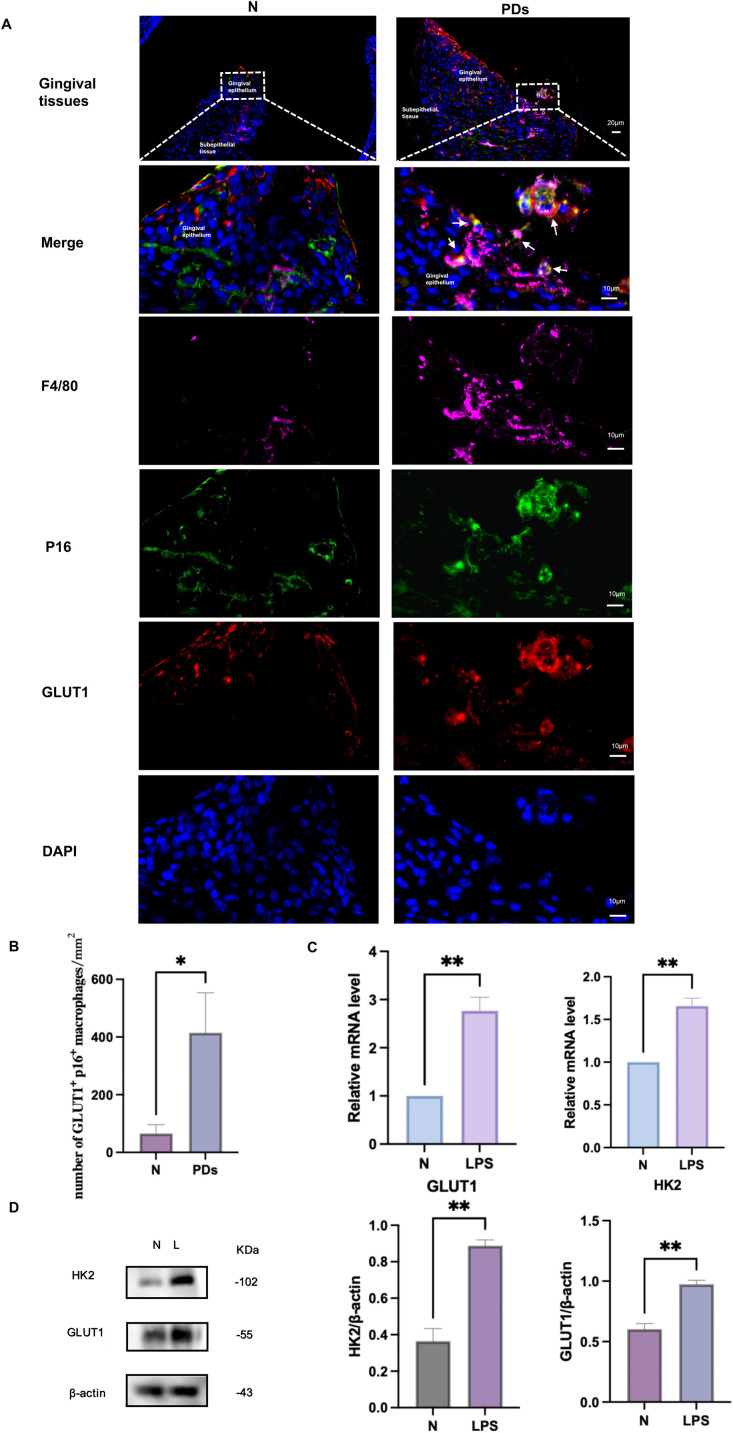
Macrophage senescence is accompanied by enhanced glycolytic expression in vivo and in vitro. A, An F4/80 antibody (pink), a p16 antibody (green), and a GLUT antibody (red) were used to stain gingival tissues of the N group and PDs group for immunofluorescence. The nuclei were stained with DAPI (blue). White arrows point to the triple-positive cells.Scale bar, 20 μm and 10 μm. B, Quantification of CSF-1R+ p16+ F4/80+cells. C, Effect of *Pg*-LPS on the expression of GLUT1,HK2 mRNA in RAW264.7 cell. D, Effect of *Pg*-LPS on the expression of GLUT1,HK2 protein in RAW264.7 cell. N, normal mice; PDs, periodontitis mice; N, control cells; L/LPS, RAW264.7 cells cultured in *Pg*-LPS(1μg/ml) for 24h. Data were represented as the mean ± SD (n=3) and analysed relative to the control group, *P < .05, **P < .01, ***P < .001, ****P < .0001 vs N

To examine how macrophage senescence is related to glycolysis, we examined glycolytic changes in gingival tissues by labelling GLUT1, a key glucose transporter and rate-limiting enzyme in glycolysis. Immunofluorescence staining revealed a notable increase in GLUT1^+^ p16^+^ F4/80^+^ triple-positive cells in the gingival tissues of mice with periodontitis compared to healthy controls. (Figure 4A,B), suggesting a connection between macrophage senescence and glycolysis in periodontitis. We further confirmed this association through cell experiments. In addition to elevated senescence-related indicators, the *Pg*-LPS group exhibited increased protein and mRNA expression of key glycolytic markers, including GLUT1 and HK2, in comparison with the control group (Figure 4C, D). In vivo and in vitro outcomes consistently reveal a robust link between macrophage senescence and glycolysis within the periodontal inflammatory microenvironment.

### Glycolysis regulated *Pg-*LPS-induced RAW264.7 cell senescence

**Fig. 5:**
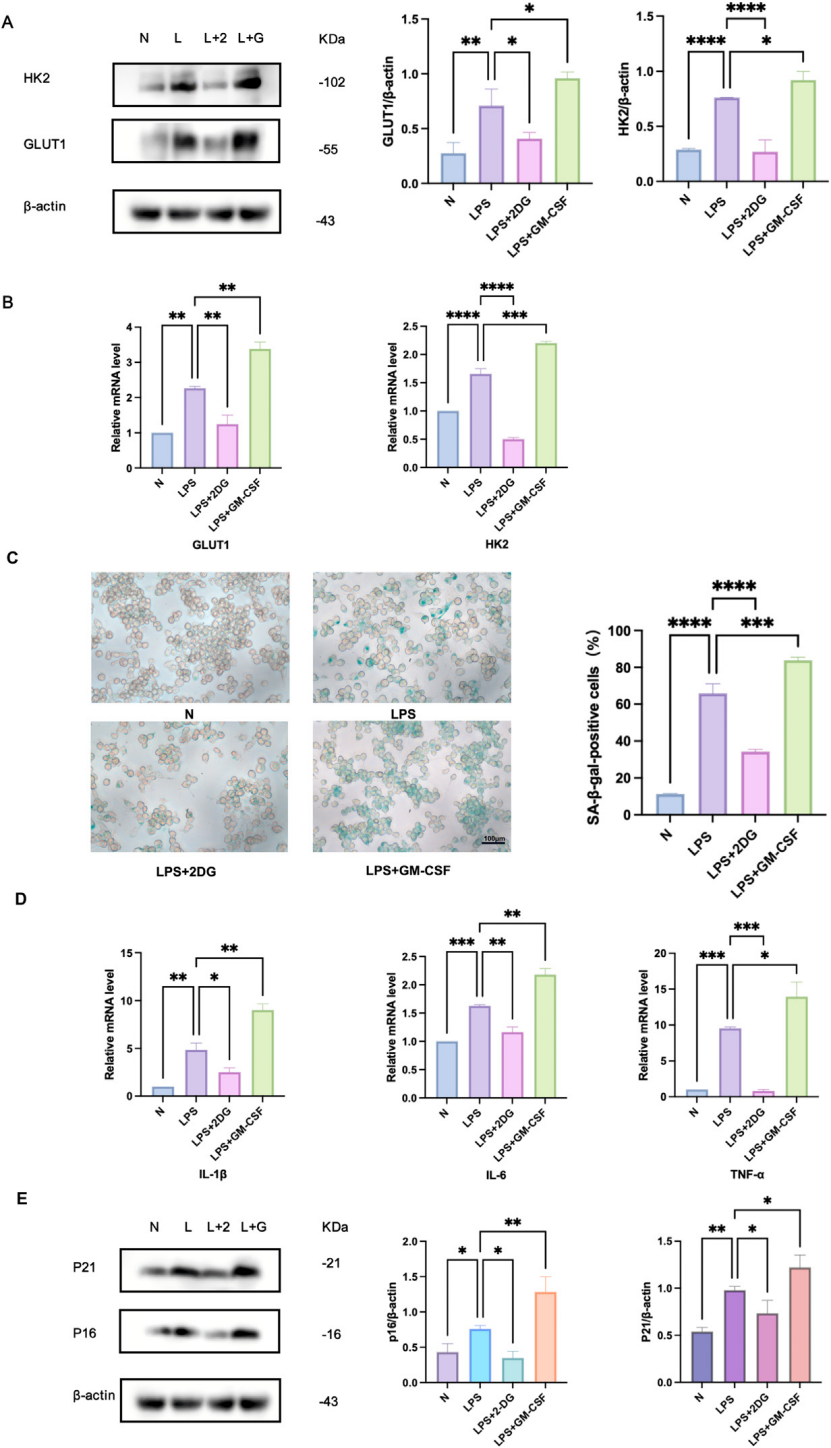
Glycolysis modulates *Pg*-LPS-induced senescence in RAW264.7 cell. A, Impact of 2-DG/GM-CSF pretreatment on HK2,GLUT1 protein expression in RAW264.7 cells stimulated by *Pg*-LPS. B, Effect of 2-DG/GM-CSF pretreatment on the expression of HK2, and GLUT1 mRNA in RAW264.7 cells stimulated by *Pg*-LPS. C, The senescent cells were visualised using SA-β-Gal staining. Senescent RAW264.7 cells were symbolised by blue. Scale bar, 100 μm. D, Effect of 2-DG/GM-CSF pretreatment on the expression of SASP factor mRNA in RAW264.7 cells stimulated by *Pg*-LPS. E, Effect of 2-DG/GM-CSF pretreatment on the p16, p21 protein in RAW264.7 cells stimulated by *Pg*-LPS. N, control cells; L/LPS, RAW264.7 cultured in *Pg*-LPS(1μg/ml) for 24h; L+2/LPS+2-DG, 1mM 2-DG pretreatment group; L+G/LPS+GM-CSF, 100ng/ml GM-CSF pretreatment group. Data were represented as the mean ± SD (n=3) and analysed relative to the *Pg*-LPS group, *P < .05, **P < .01, ***P < .001, ****P < .0001vs. LPS

To investigate the regulatory role of glycolysis in *Pg*-LPS-induced macrophage senescence, RAW264.7 cells were pretreated with 2-DG (1 mM) ^34^ or GM-CSF (100 ng/mL) ^35^ before *Pg*-LPS (1 μg/mL) stimulation (Figure 5). As shown in Figure 5A, 2-DG pretreatment significantly reduced the protein expression of the glycolytic markers HK2 and GLUT1 compared to the *Pg*-LPS group, whereas GM-CSF increased their levels. Similar trends were observed at the mRNA level (Figure 5B), with 2-DG suppressing HK2 and GLUT1 and GM-CSF elevating their expression.

Cellular senescence was assessed via SA-β-Gal staining (Figure 5C). Compared with the *Pg*-LPS group, 2-DG significantly reduced the number of SA-β-Gal-positive cells, while GM-CSF increased their proportion. Furthermore, 2-DG downregulated the mRNA levels of SASP factors (IL-1β, IL-6, and TNF-α), while GM-CSF amplified their expression (Figure 5D). Western blot analysis (Figure 5E) confirmed that 2-DG suppressed the senescence-associated proteins p16 and p21, whereas GM-CSF upregulated both markers.

These data demonstrate that glycolysis activation is both necessary and sufficient to drive macrophage senescence in the periodontal inflammatory microenvironment.

### PLX3397 inhibited macrophage senescence through glycolysis

**Fig. 6:**
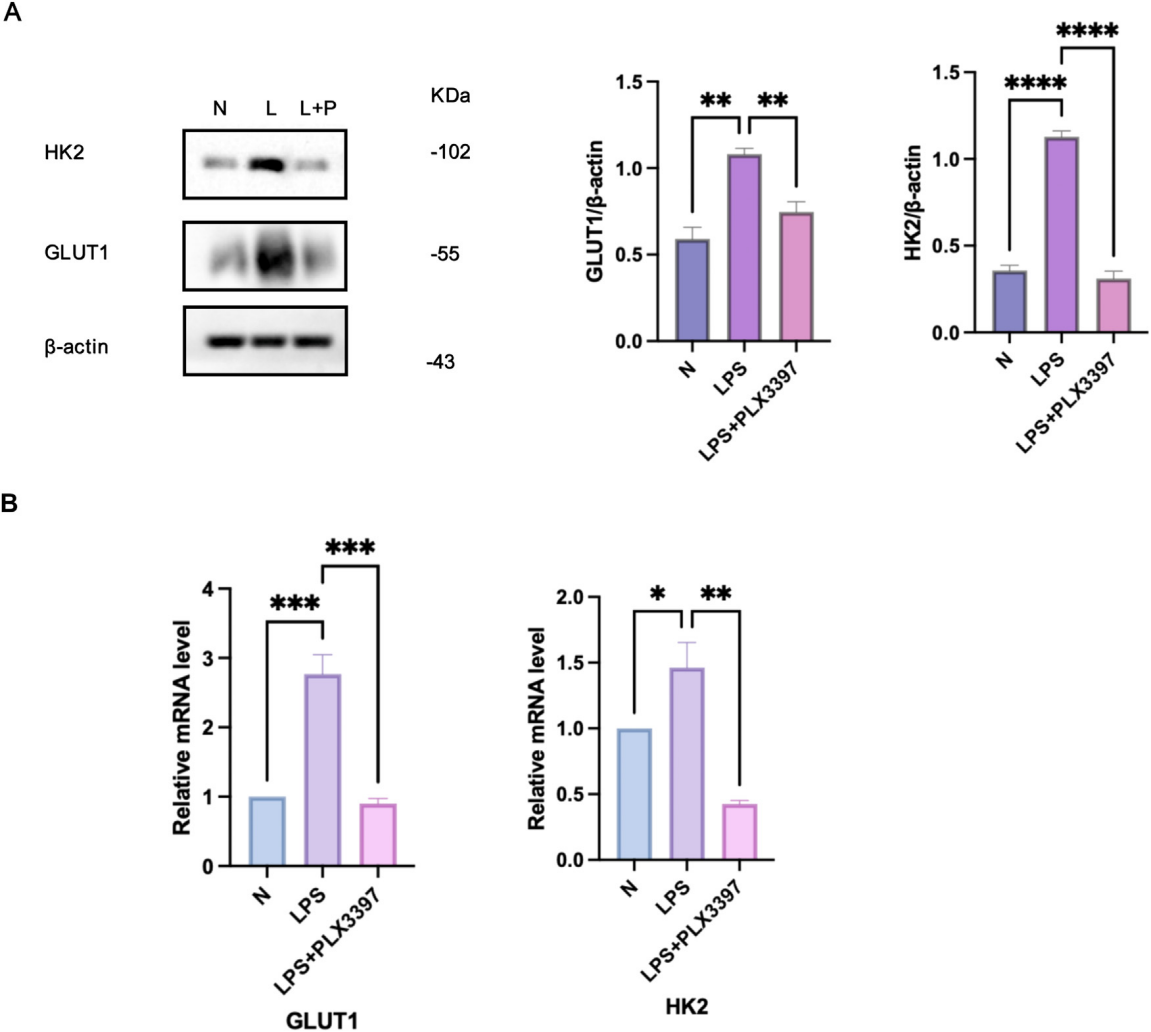
PLX3397 Inhibited RAW264.7 cell senescence through glycolysis. A.Impact of PLX3397 pretreatment on the expression of GLUT1,HK2 protein in RAW264.7 cells stimulated by *Pg*-LPS. B. Effect of PLX3397 pretreatment on the manifestation of GLUT1, and HK2 mRNA in RAW264.7 cells stimulated by *Pg*-LPS. N, control cells; L/LPS, RAW264.7 cultured in *Pg*-LPS(1μg/ml) for 24h; L+P/LPS+PLX3397,500nM PLX3397 pretreatment group. Data were represented as the mean ± SD (n=3) and analysed relative to the *Pg*-LPS group, *P < .05, **P < .01, ***P < .001, ****P < .0001vs. LPS

To investigate whether CSF-1R inhibition alleviates macrophage senescence through glycolytic regulation, RAW264.7 cells were pretreated with 500 nM PLX3397 (a CSF-1R inhibitor) prior to stimulation with 1 μg/mL *Pg*-LPS. As shown in Figure 6A, PLX3397 pretreatment significantly reduced the protein expression of glycolytic markers GLUT1 and HK2 compared to the *Pg-*LPS group. Consistently, the mRNA levels of both markers were also suppressed (Figure 6B). These results confirm that CSF-1R blockade by PLX3397 attenuates glycolytic activity in macrophages.

**Fig. 7:**
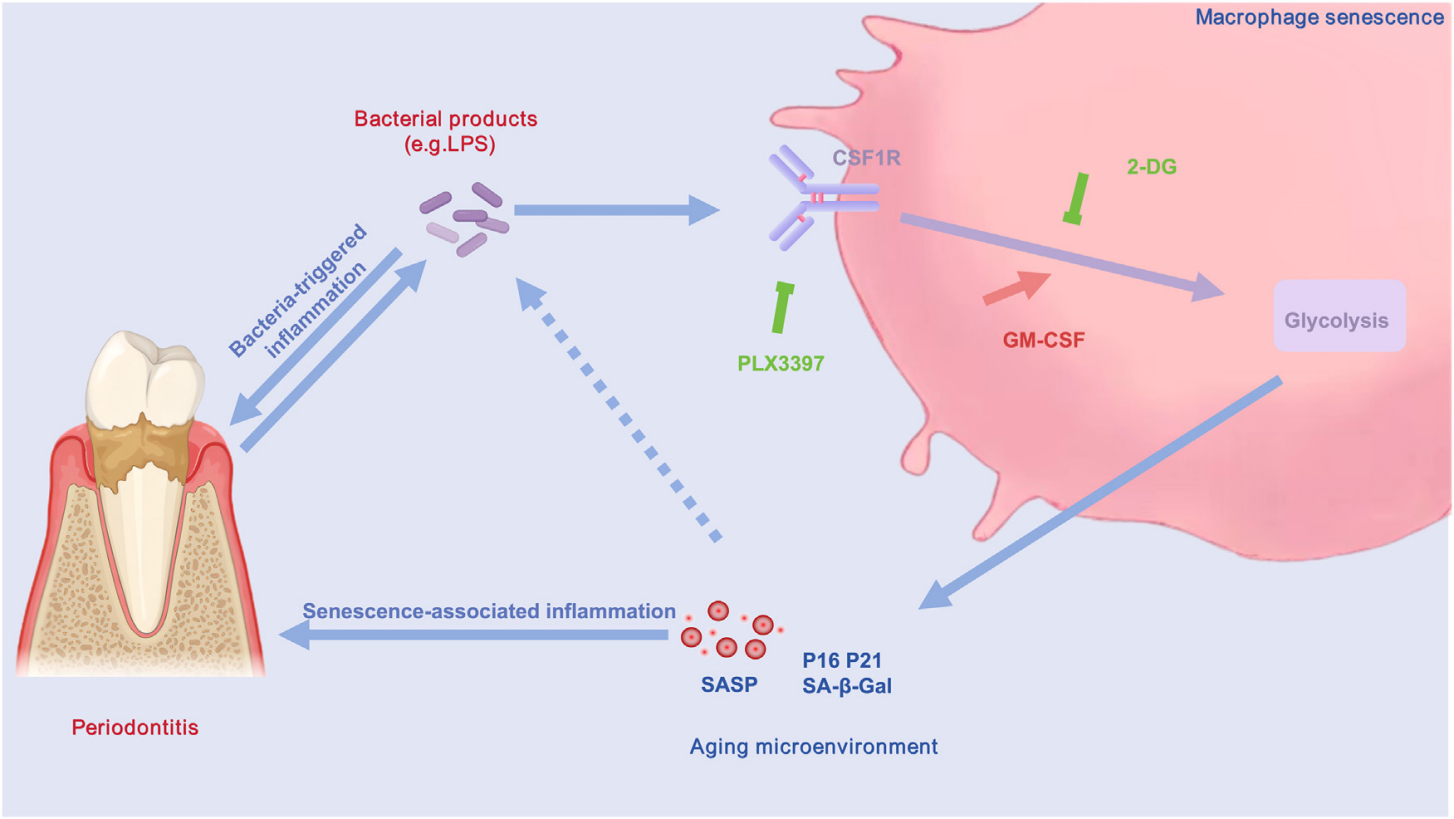
The mechanistic model demonstrates that within the inflammatory environment of periodontitis, PLX3397 mitigates macrophage senescence via the glycolytic pathway, thereby ameliorating the progression of periodontal inflammation.

When combined with our earlier findings demonstrating PLX3397-mediated reduction of senescence markers(e.g., SA-β-Gal, SASP factors, p16/p21), these data establish a mechanistic link between CSF-1R signaling, glycolytic reprogramming, and macrophage senescence (Figure 7). Specifically, PLX3397 mitigates macrophage senescence via the glycolytic pathway, thereby ameliorating the progression of periodontal inflammation.

## Discussion

The development of chronic periodontitis involves multiple factors, including inflammation from bacteria and the presence of senescent cells.^3^^,^^4^ Previous studies have suggested that PLX3397 may alleviate periodontitis by inhibiting macrophage senescence,^13^ however, its precise mechanisms are still unexamined. This research shows a strong link between macrophage senescence and periodontitis progression. Furthermore, our results highlight the pivotal function of CSF-1R in regulating macrophage glycolytic reprogramming, as inhibition of CSF-1R with PLX3397 effectively reduced glycolytic activity and ameliorated macrophage senescence.

The buildup of senescent cells is driven by 4 interconnected factors: enduring Gram-negative bacterial infection, long-lasting inflammation, ongoing tissue regeneration, and bacteria-induced local immunosuppression.^11^ Among these, macrophages play a pivotal role by influencing biofilm pathogenicity, adaptive immunity, and tissue damage. Prolonged exposure to low levels of inflammatory stimuli, such as LPS, induces macrophage phenotypic changes and triggers stress-induced senescence.^13^^,^^39^ The buildup of senescent macrophages leads to the emission of SASP factors, weakening the gingival immune barrier and exacerbating alveolar bone resorption. Consequently, interventions targeting macrophage senescence or SASP production could represent promising therapeutic strategies for periodontitis.^12^^,^^13^^,^^40^ In previous work, we used LPS to stimulate bone marrow-derived macrophages (BMDMs) to simulate the periodontal inflammatory environment in vivo and observed upregulation of senescence markers.^13^ This suggests that prolonged inflammatory stimulation leads to stress-induced macrophage senescence and elevates the number of senescent cells that accumulate locally in periodontal tissues. In this study, the periodontal tissues of mice suffering from periodontitis showed higher levels of senescence markers. *Pg*-LPS-stimulated macrophages exhibited senescence hallmarks, such as heightened SA-β-Gal activity, upregulated senescence markers, and elevated SASP factors. These outcomes highlight the role of macrophage senescence as an inflammatory amplifier, wherein senescent macrophages continuously release SASP factors, exacerbating inflammation, accelerating an increase in senescent cells, and promoting periodontal disease progression. This aligns with previous research and highlights the feasibility of targeting senescent macrophages as a strategy for periodontitis therapy.

Clinical studies have established the involvement of CSF-1R in the inflammatory progression of various types of periodontitis, including chronic periodontitis,^16^^,^^41^, ^42^, ^43^, ^44^ aggressive periodontitis,^45^ and diabetic periodontitis.^46^ CSF-1R is crucial in managing the periodontal inflammatory microenvironment, as supported by numerous studies. In the periodontal inflammatory microenvironment, CSF-1R exacerbates inflammation, promotes the release of inflammatory factors, and contributes to alveolar bone resorption.^17^^,^^41^ Inhibiting CSF-1R has been shown to mitigate inflammatory responses in gingival tissues.^41^ Additionally, in LPS-induced experimental periodontitis, CSF-1R blockade has demonstrated an osteoprotective effect by reducing osteoclast formation and alveolar bone resorption.^18^ PLX3397, a selective tyrosine kinase inhibitor of CSF-1R, is already being used in clinical settings to treat giant cell tumors in the tendon sheath that cause symptoms.^47^^,^^48^ Our research that mice with periodontitis exhibited higher expression of CSF-1R^+^ p16^+^ macrophages in periodontal tissues, and there was an increase in CSF-1R expression in RAW264.7 cells when stimulated by *Pg-*LPS. Consistent with earlier findings,^13^ our in vivo and ex vivo results confirmed high CSF-1R expression in the periodontal inflammatory microenvironment. To delve deeper into how CSF-1R inhibition might reduce macrophage senescence, we pre-treated the *Pg*-LPS-induced macrophage senescence model with PLX3397. The findings demonstrate that PLX3397 significantly inhibited the levels of SASP-associated factors, senescence markers, and SA-β-Gal activity. These outcomes suggest that targeting CSF-1R could slow macrophage senescence, reduce SASP-associated inflammatory responses, and alleviate periodontal inflammation. Collectively, these results underscore the promise of PLX3397 as a treatment approach for periodontitis through immune intervention. Thus, further studies on the mechanism by which PLX3397 inhibits macrophage senescence could aid in creating new treatment methods for periodontitis via immune intervention.

CSF-1R is essential for the reprogramming of cellular glycolytic metabolism, promoting enhanced glycolysis.^20^^,^^21^ Glycolysis is essential for cellular energy synthesis and immune cell activation. Under normal conditions, cells rely on oxidative phosphorylation (OXPHOS) to produce 32 ATP units per glucose molecule. However, under hypoxic conditions, glycolysis predominates, generating only 2 ATP units.^49^ Interestingly, tumour cells exhibit a similar metabolic shift, favoring glycolysis even in aerobic environment, a phenomenon referred to as the "Warburg effect".^50^ Such metabolic adaptation has also been observed in LPS-stimulated macrophage.^51^ Macrophages, as sentinel cells, rapidly adapt their metabolism to environmental stimuli, utilising versatile pathways for activation and energy production. This metabolic reprogramming is crucial for their activation and function.^49^ Studies suggest that metabolic reprogramming is closely linked to cellular senescence.^24^, ^25^, ^26^ Our study identified elevated expression of GLUT1^+^p16^+^ macrophages in the periodontal tissues of Periodontitis mice. Similarly, *Pg*-LPS stimulation of RAW264.7 cells elevated the levels of key glycolytic markers GLUT1 and HK2, along with a significant rise in senescence-related indicators. These findings suggest that macrophage senescence in periodontitis is closely associated with glycolysis. To further explore this connection, we manipulated glycolysis in RAW264.7 cells using GM-CSF (enhancer) and 2-DG (inhibitor). The results revealed corresponding changes in glycolytic and senescence markers, highlighting the role of glycolysis in regulating macrophage senescence. Aberrant glycolysis activation may serve as a critical trigger for macrophage senescence. Thus, targeting excessive glycolysis could alleviate the burden of senescent macrophages, improve the local inflammatory environment, and potentially offer therapeutic avenues to hinder periodontitis progression and promote tissue repair.

Our results demonstrate that *Pg*-LPS stimulation elevated glycolytic markers and senescence indicators in macrophages, establishing a link between glycolysis and macrophage senescence in the periodontal inflammatory microenvironment. Additionally, CSF-1R inhibition via PLX3397 reduced senescence markers in macrophages. Based on these findings, we hypothesised that CSF-1R influences macrophage senescence by modulating glycolytic pathways. To test this hypothesis, we conducted glycolysis index assays following CSF-1R inhibition. The findings showed a notable reduction in the expression of GLUT1 and HK2, confirming that CSF-1R inhibition suppresses glycolysis. This suggests that CSF-1R is crucial in controlling macrophage metabolism, contributing to the senescence process by promoting glycolysis under inflammatory conditions. Furthermore, PLX3397 treatment concurrently reduced glycolytic activity and senescence markers, indicating that inhibiting CSF-1R disrupts the metabolic reprogramming driving macrophage senescence. These results reinforce the connection between glycolysis and senescence while underscoring the critical regulatory role of CSF-1R in this pathway. These results align with the broader concept of metabolic interventions as a means to counteract chronic inflammation and cellular senescence.^52^

To establish the causal link between CSF-1R signalling and macrophage senescence, we employed 2 complementary approaches: (1) Pharmacological inhibition of CSF-1R using PLX3397, a clinically approved agent that functions by inhibiting the tyrosine kinase activity of CSF-1R, effectively blocking the signalling pathways^48^^,^^53^ and (2) metabolic pathway-specific intervention via combined GM-CSF/2-DG treatment to bidirectionally modulate glycolysis, this multi-level strategy revealed a tightly coupled "CSF-1R inhibition → glycolytic suppression → senescence alleviation" axis. PLX3397 treatment effectively inhibited CSF-1R, leading to suppressed glycolysis (reduced GLUT1 and HK2 levels) and decreased senescence markers. Notably, GM-CSF-induced glycolytic augmentation partially rescued senescence in PLX3397-treated cells, confirming glycolysis as a nonredundant effector downstream of CSF-1R. These findings align with mechanistic studies demonstrating CSF-1R’s direct control over myeloid cell metabolism,^20^^,^^21^^,^^54^^,^^55^ while our study extends these paradigms by positioning glycolysis as a causal bridge between CSF-1R and senescence. While genetic CSF-1R knockout models could further refine mechanistic granularity, our approach emphasises clinical translatability: PLX3397 is clinically used for CSF-1R-driven pathologies,^56^ and its combined use with metabolic modulators (2-DG/GM-CSF) lays groundwork for immunometabolic therapy development in chronic inflammatory disorders.To further elucidate mechanistic hierarchies, future work will prioritise CSF-1R manipulation models (inducible knockouts or overexpression). Such studies will complement our translational findings with deeper molecular insights.

This study provides evidence that CSF-1R inhibition alleviates macrophage senescence by suppressing glycolysis in the context of periodontitis. This mechanistic insight could guide the development of metabolism-targeted senolytic therapies. However, certain limitations should be acknowledged. First, the reliance on RAW264.7 cells and mouse models may not fully recapitulate human periodontal pathophysiology. Future studies should validate these mechanisms in primary human macrophages or clinical samples. Secondly, while PLX3397 effectively reduced senescence markers, its long-term safety and potential effects on periodontal tissue regeneration remain to be evaluated. Finally, the downstream metabolic consequences of glycolysis inhibition (e.g., lactate production and histone lactylation) were not explored, warranting further investigation.

The interplay between CSF-1R signalling and osteoclast activity in periodontitis represents a critical area for future research. Additionally, whether CSF-1R modulates lactylation-mediated epigenetic changes downstream of glycolysis also requires clarification. Although PLX3397 shows therapeutic potential, its clinical efficacy and optimal dosing regimen in periodontitis patients need rigorous validation through preclinical and clinical trials. Targeting senescent macrophages via metabolic reprogramming may synergise with existing anti-inflammatory therapies, offering a novel combinatorial approach for periodontitis management.

## Conclusion

In summary, this study demonstrates that macrophage senescence is a pivotal driver of periodontal inflammation. PLX3397 alleviates macrophage senescence by modulating glycolysis, uncovering a mechanistic link between CSF-1R signalling, immune metabolism, and senescence in periodontitis. These findings provide a foundation for developing targeted therapies to eliminate senescent cells and restore periodontal homeostasis.

## Author contributions

**Jifan Zhan:** Conceptualisation, Data curation, Validation, Writing – original draft, Writing—review and editing. **Jiabing Kan:** Formal analysis, Methodology, Supervision, Writing—original draft, Validation. **Yan Wei:** Formal analysis, Writing—original draft. **Tianjiao Xiao:** Data curation, Writing—review and editing. **Hui Fang:** Data curation, Writing—review and editing. **Yiting Yuan:** Visualisation, Writing—original draft. **Jie Zhang:** Writing—review and editing. **Li Li:** Writing—review and editing. **Yongchun Zhang:** Writing —review and editing. **Ai Tian:** Writing—review and editing, Conceptualisation, Funding acquisition, Methodology, Project administration, Supervision. All authors have read and agreed to the published version of the manuscript.

## Funding

This project was supported by the 10.13039/501100001809National Natural Science Foundation of China (82260193), 10.13039/501100005329Natural Science Foundation of Guizhou Province (Basic ZK[2024] -General 251) and 10.13039/100017957Health Commission of Guizhou Province (gzwkj2024-193).

## Ethical approval

This study was approved by the Animal Ethics Committee of Guizhou Medical University (Approval No: 2200991) and the animal experiments were followed strictly by the approved guidelines.

## Declaration of generative AI and AI-assisted technologies in the writing process

During the preparation of this work the authors used QuillBot in order to improve the language. After using this tool, the authors reviewed and edited the content as needed and took full responsibility for the content of the publication.

## Conflict of interest

The authors declare that they have no known competing financial interests or personal relationships that could have appeared to influence the work reported in this paper.

## Appendix Supplementary materials

Image, application 1

## Footnotes

Supplementary material associated with this article can be found in the online version at doi:10.1016/j.identj.2025.100843.
