# Supplementary material for: Pexidartinib Inhibits Macrophage Senescence Through Glycolysis in Periodontitis Microenvironment
Source: Int Dent J. 2025 Jun 2;75(4):100843. doi: 10.1016/j.identj.2025.100843 (PMC12167015; doi:10.1016/j.identj.2025.100843)
Supplement: Supplementary file 1 [file mmc1.docx]

**Supplementary**

**Table 1 General characteristics (n=90)**

| Characteristic | (Mean+SD or %) | Range |
| --- | --- | --- |
| Age (years) | 42+13 | 13-67 |
| Female | 50% |  |
| Race |  |  |
| black | 21% |  |
| white | 37% |  |
| Asian | 1% |  |
| Mixed | 32% |  |
| Other | 5% |  |
| Declined to report | 4% |  |
| Ethnicity |  |  |
| Hispanic | 76% |  |
| Non-Hispanic | 23% |  |
| Declined to report: | 1.1% |  |
| Periodontal diagnosis |  |  |
| Chronic periodontitis | 70% |  |
| Aggressive periodontitis | 30% |  |
| Clinical Periodontal Variables |  |  |
| Number of teeth | 28±3 | 22 -32 |
| Percent of sites with bleeding on probing (%) | 71±0.2 | 24-100 |
| Pocket depth (PD; mm) | 3.9±0.7 | 2.9-6.5 |
| Number of sites/subject with PD > 5 mm | 57±25 | 12-156 |
| Clinical attachment level (AL; mm） | 4.1±0.9 | 2.7-6.5 |
| Number of sites/subject with AL> 5 mm | 54+30 | 10-150 |
